# Effects of Seasonal Variability on the Physicochemical, Biochemical, and Nutritional Composition of Western Peninsular Malaysia *Gracilaria manilaensis*

**DOI:** 10.3390/molecules24183298

**Published:** 2019-09-10

**Authors:** Abdul Qudus Aroyehun, Kishneth Palaniveloo, Farid Ghazali, Mohammed Rizman-Idid, Shariza Abdul Razak

**Affiliations:** 1 Nutrition and Dietetics Program, School of Health Sciences, Health Campus. Universiti Sains Malaysia, Kubang Kerian 16150, Kelantan, Malaysia; 2 Biomedicine Program, School of Health Sciences, Health Campus. Universiti Sains Malaysia, Kubang Kerian 16150, Kelantan, Malaysia; 3 Institute of Ocean and Earth Sciences, University of Malaya, Jalan Universiti, Kuala Lumpur 50603, Wilayah Persekutuan Kuala Lumpur, Malaysia

**Keywords:** *Gracilaria manilaensis*, seasonal variation, physicochemical properties, nutritional properties, mineral elements, anti-microbial, heavy metals

## Abstract

This study evaluated the effect of seasonal variation on the physicochemical, biochemical, and nutritional composition of *Gracilaria manilaensis*. Sampling was designed during the main monsoon seasons in Malaysia—the Southwest monsoon (SWM) and Northeast monsoon (NEM)—to understand the intraspecific variation (*p* < 0.05). Carbohydrates, protein, and dietary fiber were found to be higher in NEM–*G. manilaensis*, whereas a higher ash content was quantified in SWM–*G. manilaensis*. No significant differences were found in crude lipid and moisture content (*p* > 0.05). Vitamin B2 was calculated as (0.29 ± 0.06 mg 100 g^−1^) and (0.38 ± 0.06 mg 100 g^−1^) for the NEM and SWM samples, respectively (*p* < 0.05). The fatty acid profile showed the dominance of saturated fatty acids (SFAs)—palmitic acids, stearic acid, and myristic acid—while the mineral contents were found to be good sources of calcium (1750.97–4047.74 mg 100 g^−1^) and iron (1512.55–1346.05 mg 100 g^−1^). Tryptophan and lysine were recorded as the limiting essential amino acids (EAAs) in NEM *G. manilaensis*, while leucine and phenylalanine were found to be the limiting EAAs in the SWM samples. None of the extracts exhibited antibacterial properties against the screened strains. The study concluded that seasonal changes have a great effect on the biochemical composition of *G. manilaensis*.

## 1. Introduction

Food security is a global concern due to the increasing human population amid dwindling natural resources in a fragile natural environment [1]. An estimated 1 billion people currently suffer from malnutrition due to insufficient dietary energy, accessibility, and micronutrient undernourishment [2,3]. However, in a renewed interest to exploit new resources to meet the growing demand for food and value-added nutritional ingredients, marine macroalgae (commonly referred to as seaweed) seem to meet the requirements [4].

The red seaweed, phylum Rhodophyta, is abundant, with nearly 6000 species globally [5]. The genus *Gracilaria* Greville (*Gracilariales*, Rhodophyta) is the second-largest commercially important agarophyte with 160 taxonomically accepted species found distributed in tropical climates and temperate regions [6,7]. It is an important source of food and agar, exceeding the capacity of the genus *Porphyra*, *Gelidium*, and *Pterocladia* due to its tolerance to a wide range of environmental conditions [8]. *Gracilaria* species is a benthic flora, existing in a diverse range of forms with a majority inhabiting the intertidal and subtidal zones in depths of 0.5 m and 10 m [9]. However, many species are still poorly known and have very limited distribution globally [10].

Seaweed contains essential macro- as well as micronutrients, consisting of high-quality protein, dietary fiber, vitamins, minerals, phytochemicals, and lipid content rich in mono and polyunsaturated fatty acids (PUFAs), which offer protection against different neurodegenerative pathologies [11]. Seaweed is an important food component in traditional Japanese, Chinese, South Korean, and Filipino cuisines [12]. Brown algae is the most consumed (66.5%), followed by red algae (33%) and green algae (5%) [13]; however, global commercial production is low.

Seaweed is consumed as both raw or processed food [14]. However, in Western countries, raw consumption is recent due to it being culturally consumed for its polysaccharides (carrageenans, agars, and alginates)—as a stabilizer, additive, and gelling and thickening agent [15]. The polysaccharides in red seaweed contain high agarose content made of polymers with D-galactose and 3,6-anhydro-L-galactopyranose subunits, which are crucial for biofuel bioconversion [16]. Seaweeds occur in abundance along the Malaysian coasts. However, seaweed horticulture is exclusive to Sabah, mainly off the coast of Semporna, Kudat, Kunak, and Lahad Datu [17]. Currently, there is an emphasis on the production of red seaweed and development of new products with keen interest in the genus *Glacilaria*. The abundance of *Gracilaria* spp. in Western Peninsular Malaysia and knowledge of their biochemical variations might offer useful information that is important to sustain the seaweed industry.

The genus *Gracilaria* is known for its low lipid content rich in PUFAs, as well as significant levels of essential amino acids (EAAs) and non-essential amino acids (NEAAs), making them highly favorable for human health [18,19,20] and comparable to other conventional protein sources, such as meat, eggs, cereal, soybean, and milk [21]. In addition to its vast nutritional properties, the genus *Gracilaria* is also known for polysaccharides, commonly referred to as dietary fiber that is not degraded by enzymes in the gastrointestinal tract [22]. Dietary fiber such as agar and carrageenans offer beneficial health outcomes, such as increasing the volume of fecal bulk and regulating cholesterol serum levels [18,22,23]. These physiological effects might be attributed to the hydrocolloid properties of seaweeds [24]. Interestingly, these properties propel the use of *Gracilaria* in food technology to produce low-calorie food products [25] that are relevant for weight control, as well as prevention of cardiovascular and gastrointestinal diseases [26,27].

The extensive coastline and numerous islands of Malaysia provide suitable habitats for the growth of diverse seaweed flora. A total of 24 species of *Gracilaria* have been recorded [28,29], and *G. manilaensis* is one of the most prolific agarophytes. Presently, *G. manilaensis* is the only species cultivated at Lumut, Perak, as feed for abalone [29] with annual production of 200 metric tonnes (Department of Fisheries Malaysia, 2018). Reports on the nutritional properties of *Gracilaria* spp. is mostly from East Malaysia [19]. However, there is no study on the effect of seasonal variations on the chemical and nutritional properties of *G. manilaensis.* To the best of our knowledge, only the fatty acids profile of *G. manilaensis* harvested from Kuala Muda, Kedah, has been reported [30] and investigations of its biological activities have shown promising antimicrobial, antioxidant, cytotoxic, and neuritogenic properties [31,32].

The chemical composition of seaweeds varies with species, maturity stage, sampling, and ecological conditions (habitat, temperature, and season), which could either stimulate or inhibit the biosynthesis of its nutrient composition [33]. The current study sought to examine the effects of seasonal variations on the biomass composition of the red macroalgae *G. manilaensis* collected from Western Peninsular, Malaysia. The proximate and nutritional composition was fully expounded to also assess its potential application as a food source.

## 2. Results

### 2.1. Chemical Composition of Seaweeds

The seasonal variation in the proximate composition of *G. manilaensis* based on dry weight (w.w.) is summarized in Table 1. The moisture content showed no statistical significant (*p* > 0.05) differences between the two seasons. However, the proximate composition showed significant difference between the NEM and SWM samples. In general, carbohydrates, dietary fiber, and crude protein contents were most abundant in the NEM-*G. manilaensis* sample, while ash content was significantly higher in the SWM sample. No significant differences were found in crude lipid content. For gross energy, *G. manilensis* collected in November displayed a higher value at (2721.67 ± 10.69 cal g^−1^) than those harvested in August (2348.33 ± 9.87 cal g^−1^).

### 2.2. Fatty Acids Compositions

The total fatty acid profile, expressed as g fatty acids methyl esters (FAME)/100 g total fat, for NEM–*G. manilaensis* and SWM-*G. manilaensis* was (99.97 ± 0.06 g 100 g^−1^) and (99.99 ± 0.06 g 100 g^−1^), respectively (Table 2). The main SFAs of both NEM- and SWM-*G. manilaensis* were palmitic acid (C16:0), followed by stearic acid (C18:0), and myristic acid (C14:0). With respect to MUFAs, only oleic acid (C18:1) was detected in both samples, but was much higher (6.19 ± 0.32 g 100 g^−1^ DW) in SWM-*G. manilaensis*. As for the PUFAs, a small fraction of eicosadienoic acid (C20:2) was noticed only in NEM–*G. manilaensis*. Overall, SFAs made up to 97.57 g 100 g^−1^ DW of total fatty acids, followed by MUFAs at 1.47 g 100 g^−1^ DW and PUFAs at 0.97 g 100 g^−1^ DW of total fatty acids, for NEM-*G. manilaensis*. Likewise, SFAs and MUFAs were quantified as 93.8 g 100 g^−1^ DW and 6.23 g 100 g^−1^ DW of total fatty acids, respectively, in SWM-*G. manilaensis*.

### 2.3. Amino Acids Compositions

The seasonal variation in the amino acids (AAs) composition of *G. manilaensis* is summarized in Table 3. The total AAs content in the NEM and SWM samples were (16.36 ± 1.31 g 100 g^−1^ DW) and (12.24 ± 1.26 g 100 g^−1^ DW), respectively (*p* < 0.05). No significant difference was found in the non-protein nitrogenous (NPN) fraction between the NEM and SWM samples (Table 1). This showed that the NPN-fraction of *G. manilaensis*, comprising of nucleic acids, nitrate, nitrite, ammonium compounds, chlorophyll, and phycoerythrine, as well as free AA, was negligible [19,34]. All the nine essential amino acids (EAAs) and non-essential amino acids (NEAAs) were found in NEM–*G. manilaenisis*. The results differed for SWM-*G. manilaensis*, which presented only six EAAs and eight NEAAs. Similarly, the total EAAs for NEM samples was higher (2840.20 ± 0.31 mg 100 g^−1^ DW) than those (665.02 ± 0.07 mg 100 g^−1^ DW) obtained from the SWM samples (*p* < 0.05). Furthermore, total NEAAs was found to be higher for NEM-*G. manilaensis* (13517.96 ± 1.13 mg 100 g^−1^ DW) than SWM-*G. manilaensis* (11578.01 ± 1.33 mg 100 g^−1^ DW). The EAAs/total AA ratio found in the SWM and NEM samples accounted for 5.73 and 17.36 g 100 g^−1^ of the EAAs, respectively. Besides, a much higher concentration of the EAAs threonine (400.74 ± 0.29 mg 100 g^−1^ DW) > leucine (331.67 ± 0.17 mg 100 g^−1^ DW) > isoleucine (285.34 ± 0.12 mg 100 g^−1^ DW) > valine (282.21 ± 0.10 mg 100 g^−1^ DW) > Phenylalanine (282.07 ± 0.09 mg 100 g^−1^ DW) > lysine (239.69 ± 0.06 mg 100 g^−1^ DW) was found in NEM–*G. manilaensis* than in the SWM sample. In the NEAAs fraction, glutamic and aspartic acids were most abundant, accounting for (57.05–58.94 g 100 g^−1^) and (9.68–11.63 g 100 g^−1^) in the NEM and SWM samples, respectively. Further observations also indicated variation in NEAA composition, as the NEM samples contain seven AAs in the order of arginine > tyrosine > alanine > serine > glycine > cysteine > proline. In contrast, five were obtained from the SWM samples in descending order alanine > arginine > serine > glycine > tyrosine > proline.

### 2.4. Mineral Composition

The mineral concentration of the 16 elements (mg 100 g^−1^ dry wt.) analyzed in *G. manilaensis* is shown in Table 4. ICP-MS analysis detected significant levels of macroelements in the order of Ca (1750.97–4047.74 mg 100 g^−1^ DW) > K (21.05–39.21 mg 100 g^−1^ DW) > Na (1.33−7.02 mg 100 g^−1^ DW) in *G. manilaensis* (*p* < 0.05). Seaweed contains higher Na and K concentrations, but a low Na/K ratio [35]. However, the Na (1.33–7.02 mg 100^−1^ DW) and K (21.05–39.21 mg 100^−1^ DW) of *G. manilaensis* were significantly low in the samples analyzed. The Na^+^/K^+^ ratio of *G. manilaensis* (0.03 and 0.33) conforms to the WHO dietary recommendation. Moreover, the total macroelement concentration noticed in the NEM-*G. manilaensis* was found to be higher (4076.97 mg 100 g^−1^ DW) than (1793.6 mg 100^−1^ DW) the SWM samples. Alternatively, total microelement concentration (1672.02 mg 100^−1^ DW) was slightly lower in NEM-*G. manilaensis* when compared to 1753.17 mg 100 g^−1^ DW recorded for SWM-*Gracilaria* spp. The order of trace elements in NEM-*G. manilaensis* were (Fe > Mn > Zn > Cu > Cr > Co > Mo > Se). As for the SWM samples, trace elements occurred in the order of Fe > Mn > Zn > Cu > Co > Cr > Se > Mo. Fe (1512.55 mg 100 g^−1^ DW) and Zn (16.40 mg 100^−1^ DW) were found to be higher in NEM-*G. manilaenisis*. In contrast (401.81 mg 100 g^−1^ DW), Mn was found to be higher in SWM-*G. manilaensis*. Assessment of heavy metals shows Al, Pb, As, and Cd varied widely between seasons, and the order of concentrations was Al (137.98–188.83 mg 100^−1^ DW) > Pb (0.536–1.38 mg 100^−1^ DW) > As (0.46–0.58 mg 100^−1^ DW) > Cd (0.004–0.009 mg 100^−1^ DW) in descending order (*p* < 0.05). Considering FAO/WHO dietary guidelines, the concentration of Al and Pb might be toxic and therefore, to minimize exposure from dietary consumption, *G. manilaensis* can be subjected to depurination to eliminate any form of impurity.

### 2.5. Vitamin Composition (mg g^−1^), and Chlorophyll Content (µg g^−1^).

The concentration of vitamins and chlorophyll contents is shown in Table 5. In this study, vitamin B2 (riboflavin) was 0.29 ± 0.06 mg g^−1^ and 0.38 ± 0.06 mg g^−1^ for NEM- *G. manilaensis* and SWM-*G. manilaensis*, respectively (*p* < 0.05). The methanolic extracts for NEM-*G. manilaensis* presented higher chlorophyll a (9.57 ± 0.01 µg g^−1^) and chlorophyll b (5.54 ± 0.02^,^ µg g^−1^) than SWM-*G. manilaensis* with chlorophyll a (7.07 ± 0.03 µg g⁻^1^) and chlorophyll b (4.26 ± 0.02 µg g⁻^1^).

### 2.6. Physicochemical Properties

Table 6 illustrates the swelling capacity (SWC), water-holding (WHC), and oil-holding capacity (OHC) of NEM-*G. manilaensis* and SWM-*G. manilaensis*. In this study, SWC and WHC were significantly increased (*p* < 0.05, one-way ANOVA, Post Hoc Test using Duncan) as temperatures increased from 25 °C to 37 °C due to increase in solubility of the fibers and protein presence in *G. manilaensis*. SWC for the NEM and SWM samples increased from (7.15 ± 0.57 mL g^−1^ vs. 7.92 ± 0.98 mL g^−1^ DW) at 25 °C to (9.80 ± 0.06 vs. 9.91 ± 0.05 mL g^−1^ DW) after incubation at 37 °C (*p* < 0.05). Similarly, WHC for the NEM and SWM-*G. manilaensis* also showed a significant increase from (9.81 ± 0.76 vs. 9.94 ± 0.67) at 25 °C to (11.89 ± 0.37 g g^−1^ DW vs. 11.07 ± 0.57 g g^−1^ DW) at 37 °C. The mean OHC for NEM-*G. manilaensis* (1.97 ± 0.20 g oil g^−1^ DW) was significantly higher than that for SWM-*G. manilaensis* (1.59 ± 0.21 g oil g^−1^ DW) (*p* < 0.05).

### 2.7. Antimicrobial Properties

The antibacterial activity of the ethyl acetate extracts of *G. manilaensis* was done via standard disc diffusion assay against four pathogenic Gram-negative bacteria strains, including *Pseudomonas aeriginosa*, *Klebsiella pneumoniae*, *Escherichia coli*, and *Salmonella serovar typhi*, as well as two Gram-positive bacteria, strain *Staphylococcus aureus* and *Bacillus subtilis*, as described by Baur et al. [37]. However, in this experiment, all extract dilutions and their replicates were not able to inhibit the growth of bacteria.

## 3. Discussion

This study shows that the proximate and nutritional composition of *G. manilensis* changes according to seasons [38]. Being marine in nature, the moisture content of seaweed can reach up to (70–90 g 100 g^−1^) fresh weight, depending largely on the phylum and species [39,40], making them highly perishable in their fresh state a few days after harvesting. The drying process plays an important role in the preservation of seaweed products and could affect their nutritional value [41,42]. It helps reduce the water activity level, which retards microbial growth, thus ensuring the conservation of desirable qualities [43]. Similarly, in industries, drying is essential because crude extracts of wet seaweed do not gel [44]. The results obtained were similar to recent data found for *G. corticata* (8.40 ± 0.65 g 100 g^−1^ DW) and *G. edulis* (10.40 ± 0.69 g 100 g^−1^ DW) [23]. Moreover, our finding is comparable to data reported by Lorenzo et al. [45], which observed the moisture content of different edible brown seaweeds species ranging from (7.95 ± 0.06 g 100 g^−1^ DW) in dried *Bifurcaria bifurcate* to (11.23 ± 0.08 g 100 g^−1^ DW) in dried *Fucus vesiculosus*. However, another study reported lower moisture content of (5.32 ± 0.10 g 100 g^−1^ DW) for *G. changii* seaweed [19].

Crude ashes are inorganic constituents found in the cell sap of seaweed. They are an essential characteristic of seaweed, contributing between (8–40 g 100 g^−1^ DW) of the mineral constituents required for human and animal nutrition [46]. In this study, *G. manilaensis* was found to have a high percent of crude ash during the SWM in August, which is consistent with previous data that linked variation in the ash content of seaweeds to changes in environmental conditions and seasons [47,48,49]. Besides, the high crude ash content shows the presence of appreciable amounts of diverse minerals found in *G. manilaensis*. This result is also in agreement with data reported by Etemadian et al. [50], high ash content of (33.16 ± 2.45 g 100 g^−1^ DW and 33.62 ± 0.61 g 100 g^−1^ DW) for *Sirophysalis trinodis* and *Polycladia myrica*, respectively.. Similarly, previous authors have also reported a higher ash content (between 20.4 ± 0.1 g 100 g^−1^ DW and 31.7 ± 0.6 g 100 g^−1^ DW) for green, brown and red seaweed (aquaculture species) from Europe [51]. However, *G. corticata* and *G. edulis* contained a lower ash content 8.10 ± 0.49% DW and 7.36 ± 0.39% DW, respectively [23]. Similarly, a lower ash content of 8.70% was reported for *G. edulis* [18]. The crude ash content found in seaweed is higher than the common edible terrestrial vegetables—(5–10 g 100 g^−1^ DW), i.e., potatoes (10.4), carrots (7.1 g 100 g^−1^ DW), tomatoes (7.1 g 100 g^−1^ DW), and sweet corn (2.6 g 100 g^−1^ DW) [52], but comparable to the green vegetable, spinach (20.4 g 100 g^−1^ DW) (USDA, 2001).

The majority of edible seaweeds have very low crude fat content (0.3–7 g 100 g^−1^ DW), revealing a low source of nutritional energy comparable with land vegetables [53]. The fat content, as observed in *G. manilaensis*, presented no statistical differences between the NEM and SWM season. Evidence of low-fat content (less than 3 g 100 g^−1^) has been reported for most *Gracilaria* spp. [18,19]. Marinho-Soriano et al. [54] reported a similar pattern wherein the lipids content of *G. cervicornis* did not vary significantly throughout the year of harvest. Alternatively, Benjama et al. [55] reported that the lipid content of *G. tenuistipitata* spikes during the rainy season with a value of (3.6 g 100 g^−1^ DW) in December in contrast to *G. fisheri*, which had lower lipids content during the rainy season with a value of 1.7% in April. Those fluctuations are due to variations in environmental conditions such as temperature, salinity, and nutrients [56,57]. The crude lipids content presented for *G. manilaensis* was lower when compared to (7.07 ± 0.33 g 100 g^−1^ DW) and (4.76 ± 0.73 g 100 g^−1^ DW) reported for *G. corticata* and *G. edulis*, respectively [23]. Similarly, a lower range of crude lipid content (1.7 g 100 g^−1^ DW) and (3.6 100 g^−1^ DW) was found for *G. fisheri* and *G. tenuistipitata*, respectively [55]. Interestingly, crude lipid of NEM and SWM-*G. manilaensis* from Johor was higher compared to (0.175 g 100 g^−1^ DW) observed in *G. manilaensis* collected in Kedah [30]. Thus, fat content in Gracilaria can widely vary, depending on the source and species [23].

The phylum Rhodophyta contains high protein content (10–30 g 100 g^−1^ DW) [58] comparable to high-protein plant foods such as soybean and wheat [33]. In the present study, protein content showed highly significant differences (*p* < 0.05) for both samples. This result agreed with previous studies by Marinho et al. [54] and Benjama and Masniyom [59] on *Graclaria* spp., which describes that protein content varies depending on season and environment. A comparable trend demonstrating fluctuations in algal protein content was recorded for red seaweed *Palmaria palmata* [58], which showed a higher protein content of (21.9 ± 3.5 g 100 g^−1^ DW) in the winter-spring season than (11.9 ± 2.0 g 100 g^−1^ DW) found in summer-early autumn. In this study, protein content for the NEM and SWM samples were higher than *G. corticata* and *G. edulis*, which was (22.84 ± 0.87 g 100 g^−1^ DW) and (25.29 ± 0.67 mL g^−1^ DW), respectively [23], despite reports of a lower crude protein content of (6.68 ± 0.94 g 100 g^−1^ DW) and (12.57 ± 1.31 g 100 g^−1^ DW) in *G. edulis* and *G. changii*, respectively [18,19]. Regardless, the high protein content recorded in *G. manileansis* suggests that it might be considered a potential marine plant source of protein [54,55].

Plant carbohydrate content is often influenced by biomass, which suggests that macroalgal carbohydrate synthesis is related to periods of maximum growth, and increased in photosynthesis activity [9,54]. The total carbohydrate content of both the NEM and SWM samples was markedly higher than those reported for *G. edulis* (10.2 g 100 g^−1^ DW) and *G. changii* 29.44 g 100 g^−1^ DW [18,19]. Other authors also found much lower carbohydrate content 8.30 ± 1.89 and 4.71 ± 0.60 in *G. corticata* and *G. edulis*, respectively. The fluctuations in the carbohydrate content of *G. manilaensis* might be due to the influence of factors such as temperature, salinity, and nutrient concentration [9,60]. Besides being an energy source, carbohydrate content is an important nutritive component of metabolic processes in seaweed [61].

Seaweeds contain a substantial quantity of polysaccharides, which comprise of soluble and insoluble dietary fibers [62]. In this study, *G. manilaensis* presented very high dietary fiber in both seasons. On the other hand, a lower dietary fiber content was found for *G. edulis* and *G. acerosa*, which had (8.9 ± 0.62 g 100 g^−1^ DW) and (13.45 ± 1.076 g 100 g^−1^ DW), respectively. Meanwhile, markedly higher dietary fiber, 64.74 ± 0.82 g 100 g^−1^ DW and 56.54 ± 7.42 g 100 g^−1^ DW, was found in *G. changii* and *G. cervicornis* [19,54]. Dietary fiber plays a crucial role in the growth and protection of beneficial intestinal flora, which helps prevent the risks of colon cancer [34]. Overall, seaweeds contain relatively low energy density due to low crude lipid content, high protein content, as well as non-digestible polysaccharides [63]. Similarly, the gross caloric content of *G. manilaensis* was found to be low in both NEM and SWM samples.

Red seaweeds are predominantly rich in SFAs and PUFAs, which offer extensive nutritional applications in the food and feed, biotechnological, cosmetic, and pharmaceutical industries [26,64]. In this study, total fatty acids content was significantly higher than those obtained by previous authors, who found (97.03 g 100 g^−1^ DW) in *G. changii* and (11.41 g 100 g^−1^ DW) in *G. edulis* [18,19]. However, a higher proportion was reported by Neto et al. [51], who found 117.15% in *Gracialaria* spp. A fatty acid profile revealed that the most abundant fatty acids in seaweeds are palmitic, followed by stearic acid, and myristic acid. A similar finding was reported for *G. changii* as the percentage of palmitic acid and stearic acid accounted for (81 g 100 g^−1^ DW) of the total SFAs [19].

Other authors, including Khotimchenko et al. [65] and Bhaskar, Narayan et al. [64], also reported the total percentage of palmitic acid and stearic acid at (85–94 g 100 g^−1^ DW) of the total SFAs in the genus *Gracilaria* spp. In contrast, pentadecanoic acid 5.12% and palmitic acid (4.45 g 100 g^−1^ DW) was recorded as the dominant SFAs found in *G. manilaensis* [30]. In general, the presence of high palmitic acid (C16:0) composition is due to the distinct character traits of individual genera [66]. Overall, the *G. manilaensis* analyzed possesses a substantial quantity of SFAs and variation in content might be attributed to the influence of abiotic factors such as light, salinity, and nutrients; as well as seasonality [57,66,67].

In the EAAs fraction, the high level found in NEM-*G. manilaensis* might be associated with the high nutrient concentration present in water bodies during the rainy season [54,55,56,59]. A similar trend was reported for *Palmaria palmata* containing substantial levels of glutamic acid, serine, and alanine during winter and early spring, but absent in summer [58]. Specimens of *G. fisheri* and *G. tenuistipitata* collected in the rainy season had significantly higher levels of EAAs in contrast to a summer harvest [55]. Amongst the EAAs, histidine was quantified as the most limiting in NEM samples at 620.98 ± 22.99 mg 100 g^−1^ followed by threonine 400.74 ± 29.12 mg 100 g^−1^. An equally similar pattern was noticed by Sakthivel et al. [18] and Syad et al. [68], as obtained in *G. edulis* and *Sargassum wightii* with histidine levels at 3.3 ± 0.16 mg g^−1^ and 7.44 ± 0.44 mg g^−1^, respectively. Our data identified threonine and lysine as the highest EAAs in the SWM species with 231.73 ± 8.66 mg 100 g^−1^ and 129.62 ± 6.45 mg 100 g^−1^, respectively. These findings differ from other studies, which reported arginine and leucine as the most limiting AAs in *G. changii* and *Bifurcaria bifurcata* at 18.69% and 7.42%, respectively [19,45].

The EAAs/total amino acid ratio (0.05–0.17) was lower than those previously reported for *Gracilaria* spp. [23,68]. As for NEAAs, glutamic and aspartic acids were the most abundant, accounting between (57.05–58.94 g 100 g^−1^ DW) and (9.68–11.63 g 100 g^−1^ DW) in the NEM and SWM samples, respectively. The high proportion of acidic AA (aspartic and glutamic acids) compared to basic AA is a characteristic of red seaweed [21,69]. Total acidic AA in the present study was higher than the data found in *G. salicornia* and *G. changii* [19,56]. The distinctive flavor and characteristic ‘umami’ taste of seaweeds are linked to aspartic and glutamic acids [70]. In terms of protein quality, NEM- *G. manilaensis* is greater due to the presence of histidine, tryptophan, and AAs bearing sulfur quantified at (2.4 g 100 g^−1^) (sum of Met and Cys) found lacking in the SWM samples. Histidine is known to contribute to antioxidant and anti-inflammatory properties [71]. The concentration of lysine, methionine, cysteine, tryptophan, and threonine is low in plants. In seaweed, cysteine is often reported to be deficient, but, when present, they are found in low concentrations [18,72]. Estimating their chemical score is essential in other to determine protein quality [73].

The protein quality and order of the restrictive AAs of *G. manilaensis* was measured by the chemical score for each EAAs using reference protein as proposed by Food and Agriculture Organization of the United Nations (FAO)/World Health Organization (WHO)/United Nations (UNU) for humans (children from 1-3-year-old, and adults) [74]. The AAs chemical score found in *G. manilaensis* during NEM and SWM were in the range of (3.22–200.16 g 100 g^−1^) to (5.98–90.35 g 100 g^−1^), respectively (Table 3). In the NEM samples, tryptophan and lysine were the limiting AAs, whereas leucine and phenylalanine contributed to the lowest AAs score in SWM-*G. manilaensis*. A similar data was reported in edible red seaweeds *G. changii* and *Porphyra* spp. [21,75]. Methionine was the most limiting AAs (64.44 g 100 g^−1^ DW) found in *G. changii* [19]. As for the total EAAs requirement, *G. manilaensis* recorded a protein value of 41.49 mg g^−1^ and 146.59 mg g^−1^ during SWM and NEM monsoon, respectively. Therefore, consumption of *G. manilaensis* will offer a measurable quantity of the required essential amino acids.

The cell wall polysaccharides of macroalgae contain multiple functional groups, i.e., anionic carboxyl, amino, sulfhydryl, sulfate, and phosphate responsible for the high complexation of metallic cation from its surroundings [76]. They accumulate most of these metals without suffering any cell damage [77]. The macro, trace, and toxic elements differ widely in seaweed [78]. In this study, Ca content is higher than the level reported by Neto et al. [51] for different seaweeds: Ulva rigida 414.3 ± 33.8 g 100^−1^ g, *Gracilaria* spp. 200.4 ± 24.3g 100^−1^ g, *Fucus vesiculosus* 1382.0 ± 5.1 g 100^−1^ g, and Saccharina latissimi 919.4 ± 32.5 g 100^−1^ g. However, lower Ca contents were reported for *G. edulis* 89.38 ± 17.87 mg 100^−1^ g and *G. changii* 625.92 ± 17.87 mg 100^−1^ g [18,19]. Seaweed is an important source of calcium, containing up to (7 g 100 g^−1^ DW) [79] and could provide an alternative source of calcium for young, pre-, post-menopausal women and expectant mothers prone to calcium deficiency disorders such as osteoporosis and preeclampsia [80].

Seaweeds are known to be rich sources of Na and K, however, with a low Na/K ratio [35]. These elements—Na (1.33–7.02 mg 100^−1^ DW) and K (21.05–39.21 mg 100^−1^ DW)—were significantly lower in both samples in comparison to the higher values found in *Gracilaria* spp [18,51]. The Na^+^/K^+^ ratio of *G. manilaensis* (0.03 and 0.33) conforms with WHO dietary recommendations. Some studies have also reported low Na/K ratios in *Gracilaria* spp [18,68] suggesting that they can help neutralize modern dietary habits that are characterized by a high intake of Na/K ratio diets [19,67]. The consumption of a high Na/K ratio diet is linked to cardiovascular diseases and early death [78].

A significant amount of trace metal was recorded in *G. manilaensis* regardless of seasonal variation, and these might be attributed to the influence of growth dynamics [81]. Studies have shown that seaweed with low biomass corresponds to a high concentration of mineral elements and vice verse [82]. The Fe (1346.05 vs. 1512.55 mg 100^−1^ g) and Mn (142.34 vs. 401.81 mg 100^−1^ g) concentration was high between the two harvest seasons. Similarly, Fe content, 1072.48 ± 20.97 mg 100^−1^ g and 557.36 ± 0.57 mg 100^−1^ g, was found to be higher in *G. corticata* and *G. edulis*, respectively [23]. Some authors reported a much lower Fe content of 436.13 mg 100^−1^g DW and 0.46 mg^−1^g DW in *G. changii* and *G. edulis*, respectively [18,19]. In general, the iron content in *G. manilaensis* is higher than most terrestrial crops (2–4 mg 100^−1^ g), such as legumes, cereal grains, nuts, and green leafy vegetables [83].

The estimated daily intakes (EDIs) of each trace metal was calculated assuming daily consumption of 8 g of dried seaweed based on a daily intake for an average adult (70 kg) in Asian cuisines [36]. In this regard, consumption of 8 g portion of *G. manilaensis* provides between (598 g 100 g^−1^ and 672 g 100 g^−1^) of the recommended daily amount (RDA) of Fe in women 19–50 years, 18 mg/day FNB / IOM (2001). Seaweed is also used as a dietary source of iron to prevent anemia caused by iron deficiency [84]. Manganese is beneficial for its metalloenzymes activities [72], and 8 g of *G. manilaensis* exceeded 100% of RDA required for adult male and female.

Studies have shown that red seaweed can accumulate heavy metals [81,85]. Based on PTWI health risk standards, daily consumption of SWM and NEM *G. manilaensis* samples (12.5 g/adult/day) contributed to (172.48 g 100 g^−1^) and (236.04 g 100 g^−1^) of Al intake, respectively. It is noteworthy that the Al concentration found in *Gracilaria* spp. during the two harvest seasons is well above the specified provisional tolerable weekly intake (PTWI) of 1 ppm of body weight (BW) [86,87]. These values were higher than those recorded by Larrea-Marín et al. [88] for *Porphyra* spp. (15.0 ± 2.55–220.8 ± 7.95 µg g^−1^ DW) and *Laminaria* spp. (32.3 ± 0.91–580.0 ± 22.10 µg g^−1^ DW) from Europe and Asia. The high Al content in *G. manilaensis* may be due to acidification of the surrounding soils in the area of harvest [89,90].

The growing number of industries in Shoal, Johor, might be responsible for these elements entering the surrounding aquatic bodies [91,92,93]. Al toxicity may lead to pathophysiology of several neurodegenerative syndromes, i.e., Alzheimer, Parkinsonism, and dementia [94]. The level of Cd in NEM and SWM samples contributed less than 2% of PTWI, suggesting no health risk to potential consumers. The concentration of Pb and total As in both samples contributed between (25–70 g 100 g^−1^) of PTWI. In particular, large dietary consumption of Pb content might lead to health complications in the nervous system and other vital organs, such as kidney cancer and blood disorders [95]. The assessment of As toxicity depends on its chemical form; they exist in different forms, such as arsenic (III), arsenic (V) or other derivatives; arsenocholine, arsenobetaine, and arsenosugars [96]. Interestingly, about (60 g 100 g^−1^) of the total As concentration is lost during processing activities [97].

Vitamin analysis revealed vitamin B in a measurable concentration, while the other vitamin components were below the detection level. In contrast, other authors reported measurable quantities of different vitamins component in genus *Gracilaria* spp. [18,68]. The chlorophyll contents detected in *G. manilaensis* is low and in agreement with studies conducted by other authors [18,68], who found that chlorophyll a and b of *Gracilaria* spp. range from (3.06 ± 0.28 µg g^−1^ vs. 1.583 ± 0.049 µg g^−1^ DW) and (2.8 ± 0.08 µg g^−1^ vs. 1.896 ± 0.10 µg g^−1^ DW), respectively. Similarly, *G. edulis* and *G. corticata* also presented chlorophyll a and b in the range of (17.14 µg/g vs. 8.96 µg/g) and (8.44 µg/g vs. 7.74 µg/g), respectively [23].

Numerous studies have demonstrated the effect of temperature variations on the physicochemical properties of edible seaweed [19,23,50,55]. The SWC of *G. manilaensis* was in the range of some edible seaweeds, i.e., *Sargassum wightii* (10 ± 0 mL g^−1^ DW), *G. changii* (9.01 ± 0.06–10.91 ± 0.06 mL g^−1^ DW) and *G. edulis* (20.0 mL/g DW) [18]. Likewise, WHC indicates a significant interaction between seaweed samples and temperature. These properties could be related to the hydrophilic nature of the charged polysaccharides (agar and carrageenan) and higher neutral sugar content found in the soluble dietary fibers of red seaweed [25]. A positive correlation between water retention has previously been observed in edible seaweed [23,98]. Similarly, our results were comparable to (9.81 ± 0.55 g g^−1^–9.97 ± 0.12 g g^−1^ DW) found for *G. edulis* and (9.93 ± 0.08 g g^−1^ DW–11.59 ± 0.04 g g^−1^ DW) reported for *G. changii* [18,19]. However, values of WHC are difficult to compare, because they depend on experimental conditions (temperature, time, and centrifugation) and sample preparation [99,100]. The higher values of WHC in *G. manilaensis* indicate they might be used as a functional ingredient for producing low-calorie food such as snacks, corn flakes, crackers, and cookies [100]. Oil holding capacity (OHC) is also a functional property, and food ingredients with high OHC values allow for the stabilization of food emulsions and high-fat food products. In this study, there were variations in OHC between the seaweed samples, which might be due to the physical entrapment of oil by capillary attraction [101]. The OHC in *G. manilaensis* was comparable to other *Gracilaria* spp. [18,19,55]. The hydrophobicity of proteins also plays a significant role in fat absorption [55,98]. Overall, the results of physicochemical properties confirmed that *G. manilaensis* could be consumed as a source of food ingredients, dietary fibers, and proteins.

Studies have reported the antibacterial properties of seaweed extracts [102,103,104]. In this study, extracts dilutions from *G. manilaensis* did not inhibit the growth of the bacteria screened. Arulkumar, et al. [105] related antimicrobial activity from *G. corticata* and *G. edulis* to the presence of unsaturated fatty acids, organic acids, and phenol compounds. However, the fatty acid profile of *G. manilaensis* lacked PUFAs and did not show a zone of inhibition against the tested pathogenic organism. These results are not in conformity with Deepak et al. [106] and Narasimhan et al. [107], who reported seasonal variation in the antibacterial activities of *Gracilaria* against different Gram-positive and Gram-negative bacteria. Variability in the production of metabolites in seaweed, which is linked to seasonal variation, could have affected the antibacterial properties of the studied seaweed [108]. Seaweed collected in the summer has been reported to exhibit higher physiological activities due to active metabolites, including fatty acids, phlorotannins, pigments, lectins, alkaloids, terpenoids, and halogenated compounds found in them as a form of adaptation [109]. In this experiment, the samples were collected in August and November, where water temperature was colder at 28 °C, which might attribute to the absence of activity. However, there is no data on seasonal variation of seaweed bioactivity in Malaysia for comparison. As such, further study is required to confirm these findings.

## 4. Materials and Methods

### 4.1. Biomass sampling and Preparation

The Malaysian climate is modulated primarily by the Southeast Asian monsoon with a cycle of two opposite regimes, i.e., winter monsoon [locally known as the northeast monsoon (NEM)] and the summer monsoon [locally known as the southwest monsoon (SWM)]. The SWM begins in late May and ends in late September, while the NEM commences around November and retreats in March of the subsequent year [29]. The country receives substantial rainfall all year-round, but the amount peaks during the NEM period [110]. The SWM season between brings less rainfall, with a recorded total monthly rainfall as low as 4.8 mm. According to available reports, the average annual total rainfall and evaporation were 1862 mm and 1098 mm, respectively [111]. However, the rate of evaporation is affected by temperature and cloudiness, and when it is cloudy, there is less sunshine, resulting in less solar radiation and lower temperature. The drier months have higher evaporation, while the rainy months have lower, influencing water body salinity [112,113,114]. The salinity of Malaysian waters ranges between 28 and 34 [115,116], while surface water temperature also fluctuates between 25.0 °C and 30.5 °C [117].

Specimens of *G. manilaensis* (Rhodophyta, Rhodophyceae) were collected twice by hand in August 2018 and November 2018 during the SWM and NEM, respectively, from Merambong shoal (1°20′14.53′′ N, 103°36′08.70′′ E) in the southern tip of Peninsular Malaysia in the Straits of Johor. These are periods of optimal harvesting, as they signal the end of the growth season of some seaweeds in Malaysia [118,119]. Besides, the growth of seaweeds reacts to a broad variety of ever-changing biotic and physical abiotic factors [120]. Approximately 3 to 5 kg of tissue was collected in each harvest. To prevent physiological injury during harvesting, the algae stem was not separated. Within 24 h of collection, they were transported to the laboratory in an ice-cold box containing seawater to prevent evaporation, thoroughly washed and cleaned with distilled water to remove contaminants (epiphytes, sand, and debris), and dried at room temperature [104]. Voucher specimens were identified by Prof Siew-Moi Phang and deposited at the Institutes of Earth and Ocean Sciences, University of Malaya. The dried sample was milled to powder using a Waring blender and stored airtight under −20 °C for further analysis.

### 4.2. Estimation of Moisture and Ash

Moisture content was determined gravimetrically by measuring sample (3 g) weight loss by drying in a hot air oven (Memmert UFP 600, Schwabach, Germany) at 105 °C until a constant weight was obtained (AOAC 934.01) [121]. Moisture content is expressed either as a percent of the oven-dry mass or of the as-received mass (A5TM D 2974-87). Ash content was quantified gravimetrically after incineration of dried algal material at 550 °C for 18 h using an electric muffle furnace (Barnstead Thermolyne) (AOAC 930.05) [122]. The leftover residue after incineration is ash. The ash content is expressed as a percentage of the mass of the oven-dried sample.

### 4.3. Estimation of Total Crude Protein

Crude protein percentage was quantified according to the classical micro-Kjeldahl method (N × 6.25) [121] using a Foss Kjeltec system as described by Zhou et al. [123]. Five-hundred mg of freeze-dried algae samples wrapped in nitrogen-free paper were oxidized in a long neck 250-mL digestion flask containing concentrated sulphuric acid (15 mL), and a mixture of potassium sulfate (7 g) and cupric sulfate (0.5 g). They were digested using a Gerhardt Kjeldatherm digester at 420 °C until a clear and colorless liquid was obtained, which indicates the complete breakdown of all organic matter. Neutralization, distillation, and titration were done simultaneously in a Foss Kjeltec 8400 Analyser unit, as the results were obtained in percent of dry weight. The content of non-protein nitrogen (NPN) fraction in *G. manilaensis* was obtained after precipitation of the proteins with trichloracetic acid (10%) [34]. Pure protein content resulted from the difference between crude protein and NPN contents.

### 4.4. Amino Acid Profile

The amino acid (AA) content of *G. manilaensis* was determined based on AOAC 999.13 with automatic online OPA/FMOC derivatization by RP-HPLC using a Shimadzu LC system (Shimadzu Corporation, Kyoto, Japan) equipped with an LC-20AD pump, DGU-20AS degasser and photodiode array SPD-M20A (PAD), and fluorescence RF-10AXL (FLD) detectors on line [20]. About 2 g of powdered *G. manilaensis* samples (total and free amino acid extracts) were derivatized with o-phthalaldehyde (OPA) and 9-fluorenylmethyl chloroformate (FMOC) (Sigma-Aldrich (Steinheim, Germany), agreeing to a protocol by Heems et al. [124]. The derivatized amino acid solutions were filtered through a 0.22μm microfiltration membrane, and the filtrates (20.0 μL) were injected onto the RP-HPLC system equipped with an HPLC column (Zorbax Eclipse XDB-C18 (4.6 × 250 mm) conditioned at 27 ± 0.1 °C. The OPA-3-MPA derivatives were detected by a programmable fluorimeter with excitation (λex) and emission (λem) wavelengths set at 338 and 262 nm, respectively, while the FMOC derivatives were identified at λex 260nm and λem 315 nm; wavelength change occurred at 18.5 min. The mobile phase used was a combination of 10 mM disodium hydrogen phosphate buffer: 10mM disodium tetraborate and 5 mM sodium azide adjusted to pH 8.2 eluent (A), and a mixture of acetonitrile, methanol, and water with a ratio of 45:45:10 (*v*/*v*/*v*) as eluent B, at a flow rate of 1.0 mL min^−1^. The amino acid standards mix (AAS18, Sigma Aldrich, MO, USA) was dissolved in 0.1 M hydrochloric acid and diluted appropriately to obtain a working solution with norvalin (Merck, MO, USA) used as the internal standard.

#### Chemical Score

The amino acid (AA) contents was then compared with the FAO reference pattern as described by FAO [74]. The AA score of essential amino acids (EAAs) was calculated using the following equation:

AA score (%) = (mg EAA in 1 g of test protein)/(mg EAA in 1 g of reference protein) × 100

### 4.5. Estimation of Crude Lipids

The total weight of the lipid extract was determined using the modified Folch Extraction method, as described by Gosch et al. [125]. Two-hundred milligram of the freeze-dried algae sample, weighed into a Teflon capped glass vial, was homogenized in 5 mL chloroform: methanol (2:1, *v*/*v*) mixture at 60 °C for 1 h. The mixture was filtered using a vacuum pump, while an additional 4 mL of the chloroform-methanol mixture was used to rinse both the filter paper and the vacuum flask to recover all lipids. The filtered crude extract was washed with 1.8 mL of 0.9% NaCl into a pre-weighed vial and centrifuged to enable phase separation with lower organic phase (chloroform); it contained the lipids collected and evaporated to dryness using a Speed Vac Concentrator. The total lipid content was determined gravimetrically.

#### Fatty Acid Composition

The fatty acid (FA) composition of *G. manilaensis* was quantified using Gas Chromatography (Hydrogen Flame Ionization Detector) GC FID, according to standard method AOAC 996.06, as described by Petrović et al. [126]. Inputs for the instrument were temperature injector—225 °C; carrier gas—helium; flow rate—0.75mL/min using a capillary column (Rt-2560, 100 m, 0.25 mm ID, 0.2 um df). Aliquots of the extracted lipids samples were saponified and methylated to fatty acid methyl esters (FAME Mix C4-C24 (18919 Supelco) using Boron trifluoride (BF3) in 0.5N methanolic solution against C11.0 triglycerides internal standard (Tokyo, Japan).

### 4.6. Estimation of Total Carbohydrate

Total carbohydrate content (%) was estimated by weight difference (100 − [moisture + ash + crude protein + crude lipid]) [127].

### 4.7. Estimation of Gross Calorific Value

The gross calorific value was estimated using the Isoperibol oxygen bomb calorimeter (IKA Calorimeter System C 2000 basic) standardized with benzoic acids. Two-hundred mg of dried algae samples were made into pellets, combusted in oxygen at 200 bar (2900 PSI) and a core temperature of up to 1000 °C (1800 °F). The total calories were calculated on an ash-free basis [128,129].

### 4.8. Estimation of Total Dietary Fiber

The total dietary fiber content in seaweeds was determined according to the (AOAC, 1995) enzymatic-gravimetric method AOAC 985.29 [99].

### 4.9. Estimation of Chlorophyll A and B

Spectrophotometric determination of Chlorophyll (A and B) was estimated according to a method by Rosemary et al. [23] with slight modification. One gram of freeze-dried seaweed was dissolved and homogenized in 96% methanol (Glas-col High-speed homogenizer) at 1000 rpm for one minute. The seaweed extract was centrifuged at 1000 rpm for 1 min. The filtered supernatant was centrifuged (Eppendorf Centrifuge 5810R) at 2500 rpm for 10 min and the absorbance measured at a range of 400–700 nm on a Shimadzu UV-Vis 1800 spectrophotometer. Chlorophyll A and B content was expressed as (µg/g of dry weight), calculated according to the following equations:

Chlorophyll A = 15.65 (A666) − 7.340 (A653)

Chlorophyll B = 27.05 (A653) − 11.21 (A666)

### 4.10. Estimation of Vitamin Content

Vitamin content of *G. manilaensis* was evaluated using HPLC (LC, Agilent, USA) method BS EN 14130:2003 and nutrition labelling analysis 1993 No. 992.06 with slight modification [130], based on the following conditions: column (Zorbax Eclipse -C18 (4.6 × 250 mm) and injection volume 20 µL. Mobile phase methanol: water (95:5) and 0.05 M ammonium acetate for Vit B1 and B2 set at UV 270 nm—1.0mL/min. Hexane and Isopropanol: 100 + 0.25 mL for Vit A and E set at UV 336 nm (flow rate 0.5 mL/min for B1 and B2) and UV 264 nm for D (flow rate 1.0 mL/min). For Vitamin C, the method used was BS EN 14130:2003 Mobile Phase 0.85% Orthophosphoric Acid with UV detector 265 nm and flow rate 0.7 mL/min. Standards include Retinyl acetate and Retinyl Palmitate (7.5 µg/mL), α-tocopherol (2 mg/mL), and (Vitamin A, E, C, D at 1 mg/mL).

### 4.11. Mineral and Heavy Metal Analysis

The mineral and heavy metal content was quantified using ICP-MS (Agilent 7700) with slight modification. About 0.5 g freeze-dried *Gracilaria* spp. was subjected to wet hydrolysis using a high-pressure polytetrafluoroethylene (PTFE) vessel containing 6 mL of 65% HNO_3_ and 2 mL of 35% H_2_O_2_, and digested in an Anton Paar microwave. After digestion, the samples were filtered and diluted with Milli-Q water to a final volume of 50 mL and analyzed in an Agilent 7700 series ICP-MS for multi-mineral elements, as adopted by NMKL (Nordic Committee on Food Analysis) method 22, as well as a CEN (European Normalization Organization) method EN 15763:2009.23. The total concentrations of freeze-dried seaweed minerals were then quantified from the calibration curves of their respective standard elements [131].

### 4.12. Physicochemical Properties of Seaweeds

Swelling capacity (SWC) and water holding capacity (WHC) were determined as per the method described by Sakthivel and Devi [18], while oil holding capacity (OHC) was measured according to Yaich et al. [98].

#### 4.12.1. Swelling Capacity (SWC)

SWC was measured by the bed volume technique, as per Sakthivel and Devi [18]. About 200 mg of *Gracilaria* spp. was dissolved in 20 mL of distilled water; the mixture was stirred vigorously and conditioned separately at two different temperatures—25 °C and 37 °C—separately to measure its effect on SWC. The swelling volume was measured and expressed as ml of swollen sample per g of sample dry weight (DW):

SWC = Initial volume of water (mL) − volume of water after incubation (mL)

#### 4.12.2. Water Holding Capacity (WHC)

Water absorption of *Gracilaria* spp. was measured by the centrifugation method, according to Sakthivel and Devi [18]. About 200 mg of seaweed sample was dissolved in 20 mL of distilled water and kept in an incubator shaker at RPM 250 (IKA KS 4000I) for 24 h before it was conditioned separately at two different temperatures—25 °C and 37 °C. The supernatant was discarded after centrifugation for 25 min at 3000 g and the moisture content estimated by dehydration in an oven at 160 °C for two h. WHC is expressed as the grams of water held by 1 g of dry weight of the sample.

WHC = Wet weight of the sample (g) – Dry weight of the sample (g)

#### 4.12.3. Oil Holding Capacity (OHC)

The oil holding capacity of *Gracilaria* spp. was measured according to a method described by Yaich et al. [98]. About 0.5 g of seaweed sample was dissolved in 6.0 mL of corn oil in a centrifugation tube. The tubes were agitated for 30 min in an incubator shaker (RPM 250) (IKA KS 4000I) at 25 °C and 37 °C. Then the oil supernatant was measured at 2500 g for 30 min (Eppendorf Centrifuge 5810R) at room temperature. The OHC of seaweed was measured as the number of grams of oil held by 1 g of dry weight of the sample:

OHC = Initial volume of oil (g) − Volume of oil after incubation (g)

#### 4.12.4. Antimicrobial Properties

About 1350–1500 g of freshly harvested red seaweed *G. manilaensis* was soaked separately in 7500 mL of methanol (100%) at a ratio of 1:5 (powder/solvent) for seven days. The mixture was agitated multiple times during the soaking period to ensure maximum homogeneity and extraction of macroalgae constituents. The mixtures were first filtered by cheese cloth and then by Whatman No. 1 filter paper. The filtrate was then concentrated under reduced pressure by using a rotary evaporator at 40 °C to complete dryness, yielding 12–15 g (0.8–1.0%) of crude methanolic extract [132]. The crude methanolic extract was subjected to partitioning in a separating funnel with a mixture of two solvents—water and ethyl acetate (EtOAc)—at a ratio of 3:1, and then concentrated in vacuo to produce both water and EtOAc concentrate. The *G. manilaensis* from NEM produced 1.97 g, a higher yield than *G. manilaensis* obtained from SWM (1.02 g).

The solid crude extracts (31.5–500 µg), obtained from the lyophilised *G. manilaensis* were dissolved and diluted with EtOAc solvent. Sterilised 6-mm filter paper discs loaded with 20 µL of the seaweed extract were transferred to sterile petri dishes. The discs were allowed to remain at room temperature until complete diluent evaporation and kept under refrigeration until use. The antibacterial activity was evaluated by standard disc diffusion assay against four pathogenic Gram-negative bacterial strains—*Pseudomonas aeruginosa*, *Klebsiella pneumoniae*, *Escherichia coli*, and *Salmonella serovar* typhi—as well as two Gram-positive bacterial strains—*Staphylococcus aureus* and *Bacillus subtilis*—on Mueller Hilton agar (Difco, US), as described by Bauer et al. [37]. Pure bacterial strains were obtained from the Department of Microbiology, School of Health Sciences, Universiti Sains Malaysia, and were subsequently grown at 37 °C and maintained on nutrient agar (Merck, Germany). Bacterial isolates were grown overnight in Mueller Hilton broth (Merck, Germany) at 30 °C. Before inoculation, bacterial cell density was adjusted to turbidity equalling the standard McFarland 0.5 at 600 nm. Discs loaded with extracts were placed onto the Mueller Hilton agar (MHA) containing 100 µL standardized bacterial suspension and incubated at 30 °C for 24 h under aerobic conditions. Prior to incubation, all Petri dishes were placed in the refrigerator for 40 min to retard microbial growth. Imipenem was used as a positive control, and analytical EtOAc was used as negative control. The inhibition zone was expressed as the diameter of the inhibition zone around the discs in mm after overnight incubation. Tests were performed in duplicate.

### 4.13. Statistical Analysis

All of the analyses were performed in triplicates (n ≥ 3) and the results are presented as means ± S.D. except Dietary Fiber (n ≥ 2). Paired sample *t*-test was used to compare composition values and significant difference between the mean values of NEM and SWM specimen harvested in the two seasons. One-way analysis of variance (ANOVA) and Duncan’s test was used to compare the effects of temperature on the physicochemical properties. All determinations were performed using SPSS 24.0 (Statistical Package for the Social Sciences, SPSS Inc., Chicago, IL, USA). A significant positive variation was defined at the significance level of *p* < 0.05.

## 5. Conclusions

This study discovered pronounced seasonal variation in the biochemical composition of *G. manilaensis* for future exploitation as sustainable sources of food ingredients. Currently, there is no published data on harvest time and its relation to the biochemical and nutritional composition of the edible *G. manilaensis* species in Malaysia. Regarding the biochemical composition of *G. manilaensis*, NEM samples presented the maximum values for almost all the components investigated when compared to the SWM samples, with a significant difference. The EAAs and NEAAs were significantly higher in NEM samples, as well as proximate and gross energy value. However, high ash content was observed in the SWM, which was also reflected in the total mineral content. The lipids components showed no statistically significant difference; however, the SWM samples contained higher amounts of monounsaturated oleic acid. With respect to physicochemical properties, both samples from NWM and SWM could be considered as sources of food ingredients, including proteins, and dietary and soluble fiber. In view of these results, this study suggests that NWM and SWM samples contains important nutritive components that may significantly contribute to both human and animal nutritional requirements. Furthermore, they could serve as potential sources of mineral supplements, although the presence of high levels of aluminum and lead, which is perhaps attributed largely to the presence of human activities in coastal areas, may discourage its utilization as food and feed purposes. However, the beneficial or detrimental health effects of specific nutrients available in food depend on their absorption in the gut (which is also a function of the body’s processing conditions) and interaction with other components.

## Figures and Tables

**Table 1:** Variation in Proximate Composition (g 100 g^−1^ DW) and Gross Calorific Value (cal g^−1^) Of *G. manilaensis* Collected in Johor, Malaysia.

Composition	*G. manilaensis* (NEM)	*G. manilaensis* (SWM)
**Moisture**	9.59 ± 0.40	9.06 ± 0.10
**Ash**	30.26±0.13 ^b^	38.48 ± 0.23 ^a^
**Crude Lipids**	1.20 ± 0.15	1.13 ± 0.18
**Crude Protein**	19.39 ± 0.12 ^a^	16.03 ± 0.26 ^b^
**Pure protein**	16.38 ± 0.24 ^a^	12.34 ± 0.31 ^b^
**NPN**	3.01 ± 0.50	3.69 ± 0.11
**Carbohydrate**	39.56 ± 0.26 ^a^	35.30 ± 0.34 ^b^
**Total Dietary Fiber**	31.07 ± 1.08 ^a^	22.16 ± 0.11 ^b^
**Caloric Value (cal g^−1^)**	2721.67 ± 10.69 ^a^	2348.33 ± 9.87 ^b^

NEM, samples from Northeast Monsoon (Straits of Johor). SWM, samples from Southwest Monsoon (Straits of Johor). Results are expressed as Mean ± SD (n = 3). Means with different superscripts (a,b) within the same line were significantly different (*p* < 0.05).

**Table 2:** Variation in the Fatty Acid Composition (g Fatty Acid Methyl Esters/100 g Total Fat) of *G. manilaensis*.

Fatty Acids (g 100 g^−1^ DW)	*G. manilaenisis* (NEM)	*G. manilaensis* (SWM)
**SFAs**		
**Myristic acid (C14:0)**	2.6 ^b^	3.73 ± 0.06 ^a^
**Pentadecanoic acid (C15:0)**	0.9	ND
**Palmitic acid (C16:0)**	89.27 ± 0.21 ^a^	83.87 ± 0.64 ^b^
**Heptadecanoic acid (C17:0)**	0.77 ± 0.06	ND
**Stearic acid (C18:0)**	3.97 ± 0.06 ^b^	6.2 ± 0.7 ^a^
**Total**	97.5 ± 0.05 ^a^	93.8 ± 0.15 ^b^
**% of SFA in total FAs**	97.57 ± 0.05 ^a^	93.8 ± 0.03 ^b^
**MUFAs**		
**Oleic acid (C18:1ῳ9 cis)**	1.47 ± 0.06 ^b^	6.19 ± 0.32 ^a^
**% of MUFAs in total FAs**	1.47 ± 0.03 ^b^	6.19 ± 0.18 ^a^
**PUFAs**		
**Eicosadienoic acid (C20:2 ∆11,14)**	0.97 ± 0.06	ND
**% of PUFAs in total FAs**	0.97 ± 0.03	-
**Total fatty acids**	99.97 ± 0.06	99.99 ± 0.06

Mean ± standard deviation of three replicates. Different lowercase letters within the same row indicate statistical differences between groups (*p* < 0.05). “ND” indicates “not detected”; polyunsaturated fatty acids (PUFA); monounsaturated fatty acids (MUFA); saturated fatty acids (SFA).

**Table 3:** Variation in the Amino Acids Composition (mg 100 g⁻^1^ DW) of *G. manilaensis*.

Amino acids	*G. manilaensis* (NEM)	mg g^−1^ Protein/(AA Score (g 100 g^−1^)	*G. manilaensis* (SWM)	mg 100 g^−1^ Protein/AA Score (g 100 g^−1^)	FAO/WHO/UNU (2007)
**Essential Amino acids**					
Histidine (His)	620.98 ± 0.23	32.03 (200.16)	ND	ND	16
Threonine (Thr)	400.74 ± 0.29 ^a^	20.67 (82.67)	231.73 ± 8.66 ^b^	14.46 (90.35)	25
Valine (Val)	282.21 ± 0.10 ^a^	14.56 (36.39)	96.39 ± 11.80 ^b^	6.01 (15.03)	40
Methionine (Met)	393.13 ± 0.16	20.28 (88.15)	ND	ND	23
Tryptophan (Try)	4.37 ± 0.02	0.2254*(3.22)	ND	ND	7
Phenylalanine (Phe)	282.07± 0.09 ^a^	14.55 (35.48)	85.85 ± 2.35 ^b^	5.36 (13.06)	41
Isoleucine (Ile)	285.34 ± 0.12 ^a^	14.72 (49.05)	62.90 ± 4.30 ^b^	3.92 (13.08)	30
Leucine (Leu)	331.67 ± 0.17 ^a^	17.19 (28.04)	58.51 ± 1.99 ^b^	3.65 (5.98)	61
Lysine (Lys)	239.69 ± 0.06 ^a^	12.36 (25.75)	129.62 ± 6.45 ^b^	8.09 (16.85)	48
**TEAAs**	2840.20 ± 0.31 ^a^	146.59	665.02 ± 0.07 ^b^	41.49	291
**Non-essential acids**					
Aspartic acid (Asp)	9640.82 ± 0.22 ^a^		6984.12 ± 0.18 ^b^		
Glutamic acid (Glu)	1583.21 ± 0.54 ^a^		1423.74 ± 1.10 ^b^		
Asparagine (Asn)	ND		ND		
Serine (Ser)	304.44 ± 0.18 ^a^		289.97 ± 0.16 ^b^		
Glutamine (Gln)	ND		ND		
Glycine (Gly)	216.77 ± 0.06 ^b^		364.15 ± 0.11 ^a^		
Arginine (Arg)	573.41 ± 0.27 ^b^		830.29 ± 0.15 ^a^		
Alanine (Ala)	314.29 ± 0.18 ^b^		1366.05 ± 0.12 ^a^		
Tyrosine (Tyr)	499.26 ± 0.22 ^a^		196.47 ± 0.15 ^b^		
Cysteine (Cys)	200.82 ± 0.33		ND		
Proline (Pro)	185.14 ± 0.09 ^a^		123.23 ± 0.03 ^b^		
**∑∑NEAA**	13517.96 ± 1.13 ^a^		11578.01 ± 1.33 ^b^		
**∑AA**	16358.37 ± 1.31 ^a^		12243.04 ± 1.26 ^b^		
**EAAs/Total AA**	0.17 ± 0.14 ^a^		0.05 ± 0.12 ^b^		
**∑EAA_S_/Total AAs(%)**	17.36 ± 0.14 ^a^		5.43 ± 0.12 ^b^		
**∑EAAs/∑NEAAs**	0.2101 ± 0.0021 ^a^		0.0573 ± 0.0011 ^b^		

The FAO requirement protein pattern referenced is amino acid scoring pattern for use in schoolchild/adolescent (3–10) year of age (WHO/FAO/UNU 2007). **∑** AA = Total Amino acids. (mean ± standard deviation values) (n = 3). expressed as mg/g seaweed on a dry weight basis. * EAAs: Essential amino acids; NEAAs: Non-essential amino acids. a,b values with different superscripts within the same line are significantly different.

**Table 4:** Seasonal Variation in the Mineral Composition (mg 100 g^−^^1^ DW) of Edible *G. manilaensis* as Compared With the Dietary Reference Intake Values (RNI) (8 g Day^−^^1^) of Australia.

Minerals (mg 100 g^−1^)	*G. manilaensis* (NEM)	*G. manilaensis* (SWM)	Australia (RDIs)
**Macro Metals**			
Calcium (Ca)	4047.74 (32.38)	1750.97 (14.01)	1000 mg/day
Magnesium (mg)	1.16 (0.023)	2.09 (0.042)	400 mg/day
Potassium (K)	21.05(0.06)	39.21 (0.11)	2.8 & 3.8 g/day AI
Sodium (Na)	7.02 (0.02)	1.33 (0.01)	2.3 g/day UL
Na/K	0.33	0.03	<0.49
**Total**	4076.97	1793.60	
**Trace Metals**			
Copper (Cu)	0.31 (0.25)	0.39 (0.31)	10 mg/day UL
Iron (Fe)	1512.55 (672.24)	1346.05 (598.24)	8–18 mg/day
Manganese (Mn)	142.34 (207040)	401.81 (584455)	5.0 & 5.5 µg/day AI
Molybdenum (Mo)	0.05 (8.89)	0.03 (5.33)	0.045mg/day
Selenium (Se)	0.048 (5.5)	0.057 (6.2)	60 &70 µg/day
Zinc (Zn)	16.40 (9.4)	4.42 (2.5)	0.8 &14 mg/day
Chromium (Cr)	0.26 (59.44)	0.19 (43.43)	25 & 35 µg/day AI
Cobalt (Co)	0.06	0.22	
**Total**	1672.02	1753.17	
**Heavy Metal/** **EDIs (g 100 g^−1^)**			WHO/FAO TWIs
Arsenic (As)	0.46 (38.33)	0.58(48.33)	15 µg/kg BW
Cadmium (Cd)	0.004 (0.71)	0.009 (1.61)	7 µg/kg BW
Aluminium (Al)	188.83 *(236.04)	137.98*(172.48)	1000µg/d body BW
Lead (Pb)	1.38 (69)	0.536 (26.8)	25µg/kg BW

Triplicate measurements of each sample with RSD is less than 10 g 100 g^−1^ Values indicated in () represents the approximate % of estimated daily intakes (EDIs) in comparison to the Recommended Dietary Allowances (RDA) [36]. Method Detection Limit (MDL). Nutrient reference values for Australia and New Zealand for men and women (Australia New Zealand Food Authority, 2005). Recommended Daily Intake (RDI); Tolerable Upper Intake Level (UL); Adequate Intake (AI); Body Weight (BW), provisional tolerable weekly intakes (PTWIs) (FAO/ WHO, 2011).

**Table 5:** Variation in Vitamin Composition (mg g^−^^1^ DW), Chlorophyll A and B Content (µg g^−1^ DW) of *G. manilaensis*.

Composition (mg 100 g^−1^ DW)	*G. manilaensis* (NWM)	*G. manilaensis* (SWM)
**Vitamin A (IU g^−1^)**	Below detectable level (<1)	ND (<1)
**Vitamin B1**	Below detectable level (<1)	ND (<1)
**Vitamin B2**	0.29 ± 0.06	0.38 ± 0.06
**Vitamin D (mcg 100 g^−1^)**	Below detectable level (<5)	ND (<5)
**Chlorophyll a**	1.25 ± 0.06 ^a^	1.29 ± 0.06 ^b^
**Chlorophyll b**	9.57 ± 0.01 ^a^	7.07 ± 0.03 ^b^

Results are expressed as Mean ± SD (n = 3). ^a,b^ values with different superscripts within the same line are significantly different.

**Table 6:** Variation in The Physicochemical Properties of Edible *G. manilaensis* at 25 °C and 37 °C.

Seaweed	SWC (mL g^−1^)		WHC (g g^−1^)		OHC (g g^−1^)
	**25 °C**	37 °C	25 °C	37 °C	25 °C
***G. manilaensis* (NEM)**	7.15 ± 0.57 ^bB^	9.91 ± 0.05 ^aA^	9.81 ± 0.76 ^bB^	11.89 ± 0.37 ^aA^	1.97 ± 0.20 ^a^
***G. manilaensis* (SWM)**	7.92 ± 0.98 ^aB^	9.80 ± 0.06 ^bA^	9.94 ± 0.67 ^aA^	11.07 ± 0.57 ^bB^	1.59 ± 0.21 ^b^

Values are Mean ± SEM, n = 2 on DW. ^a,b^ values with different superscripts within the same column are significantly different between seaweeds. ^A,B^ values with different superscripts within the same line shows significant differences between temperatures.
